# Supplementary material for: A novel CHCHD10 mutation implicates a Mia40‐dependent mitochondrial import deficit in ALS
Source: EMBO Mol Med. 2018 May 22;10(6):e8558. doi: 10.15252/emmm.201708558 (PMC5991575; doi:10.15252/emmm.201708558)
Supplement: Supplementary file 1 — Expanded View Figures PDF [file EMMM-10-e8558-s001.pdf]

Expanded View Figures

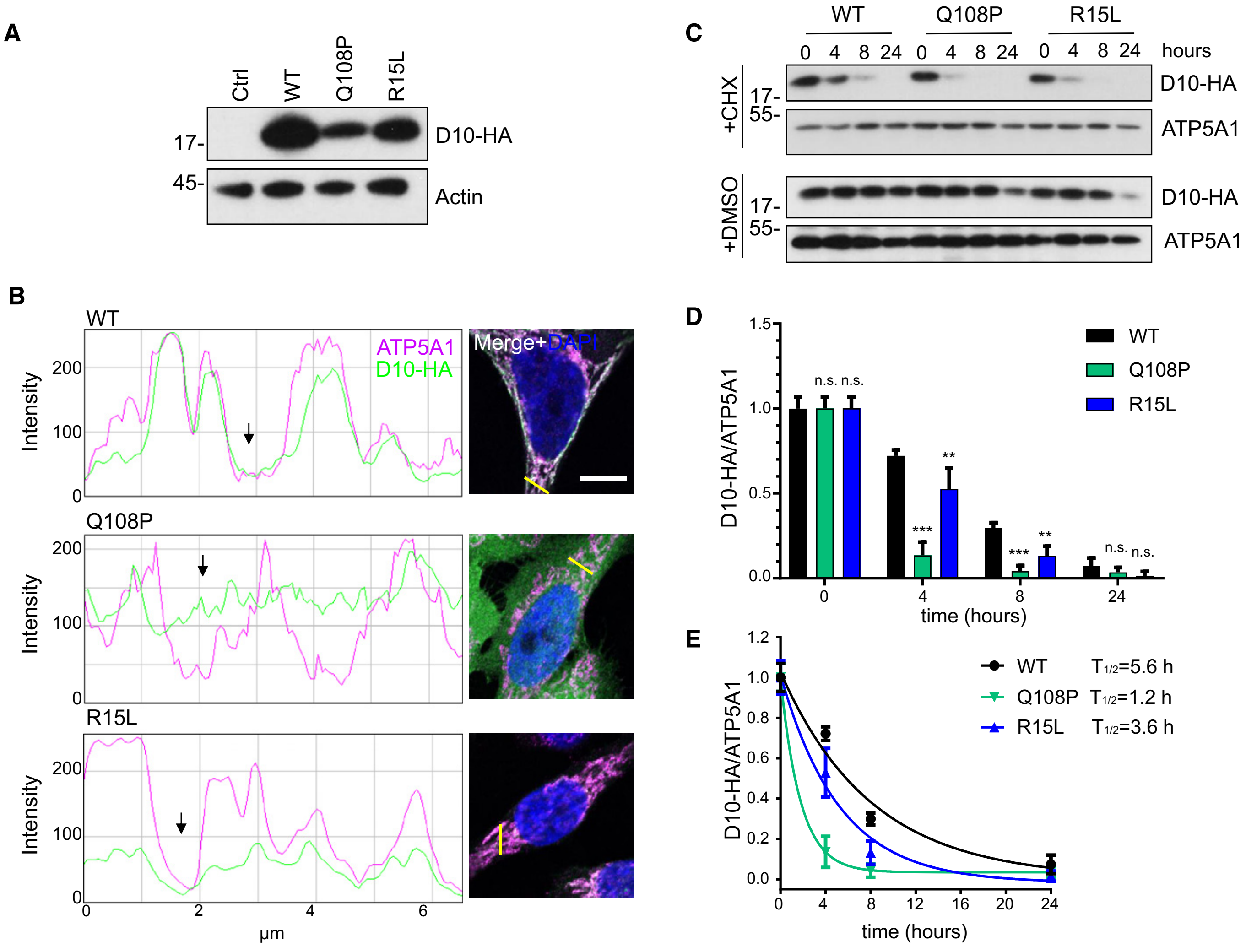

Figure EV1. CHCHD10 mutants are less stable.

A CHCHD10-HA (D10-HA) level in whole cell lysates of HeLa cells transfected with empty vector (Ctrl) or HA-tagged CHCHD10 variants (WT, Q108P, or R15L).

B Line scans of CHCHD10-HA (D10-HA) and ATP5A1 intensity in immunofluorescence pictures (right) of HeLa cells transfected with the indicated HA-tagged CHCHD10 constructs. Intensities of the red (ATP5A1, here shown in magenta) and the green (D10-HA) channels were measured along a 6.5-µm-long line (yellow), and diagrams were generated with the plugin RGB-profiler in ImageJ. Scale bar represents 10 µm.

C Protein stability of HA-tagged CHCHD10 (WT, Q108P, or R15L) was measured in HeLa cells transfected for 2 days and then treated with cycloheximide (+CHX) or vehicle (+DMSO) and harvested after 0, 4, 8, and 24 h. Note that steady state levels of CHCHD10 Q108 and R15L in DMSO-treated cells are also lower at 24 h, i.e. 3 days after transfection.

D Quantification of HA-tagged CHCHD10 (D10-HA) protein levels normalized to ATP5A1. Transfected HeLa cells were treated with cycloheximide for 0, 4, 8, and 24 h. Data are shown as mean ± SD. Two-way ANOVA (followed by Turkey's multiple comparison): $n = 3$ biological replicates, $t = 4$ h WT versus Q108P: ***$P < 0.0001$, $t = 4$ h WT versus R15L: **$P = 0.002$, $t = 8$ h WT versus Q108P: ***$P < 0.0001$, $t = 8$ h WT versus R15L: **$P = 0.0076$.

E Half-life analysis of the respective CHCHD10 variant was calculated by nonlinear regression analysis. Data are shown as mean ± SD. $n = 3$ biological replicates.

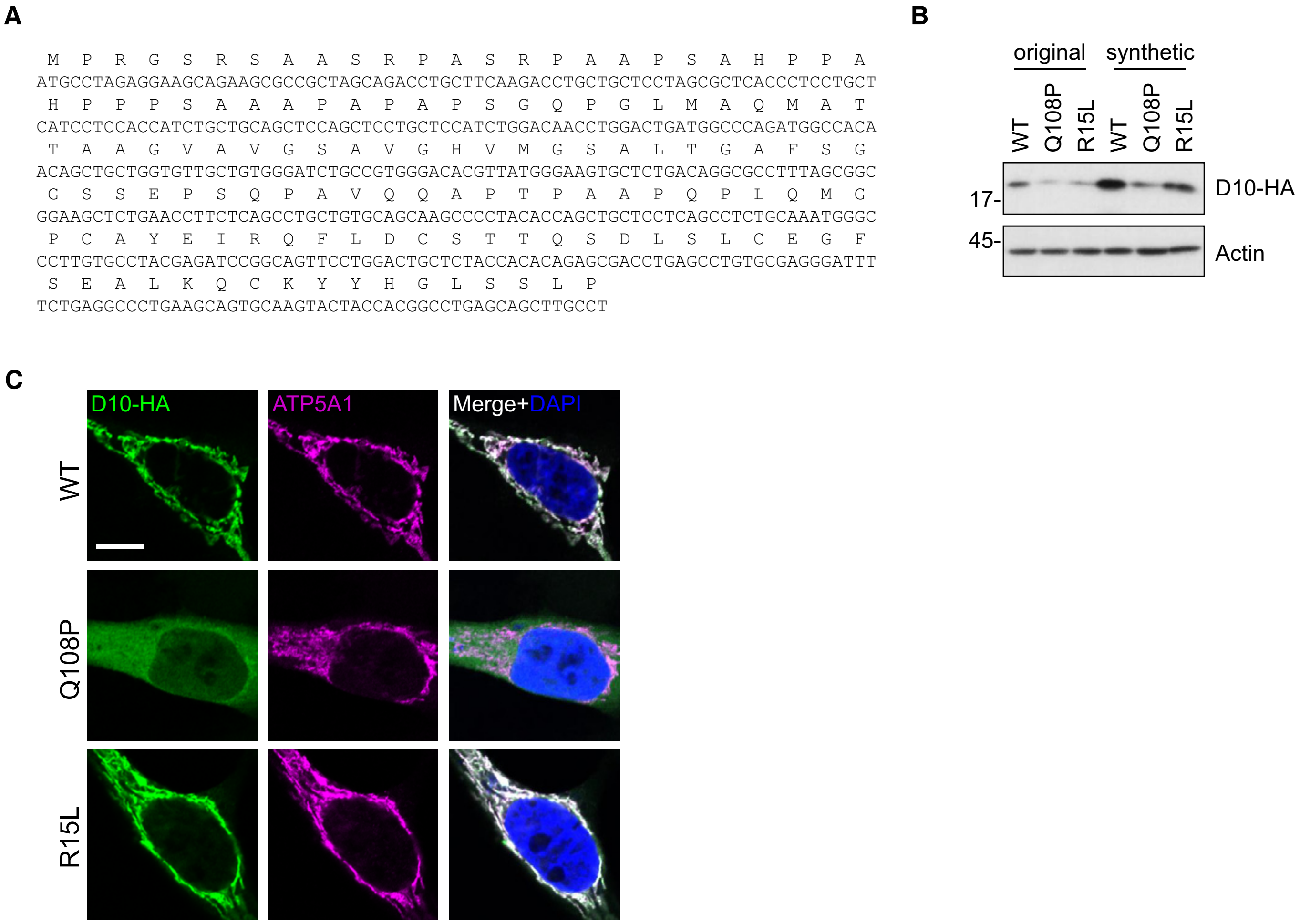

Figure EV2. Import deficit of CHCHD10 Q108P is replicated with a high-expressing synthetic gene.

A Sequence of the codon-optimized synthetic CHCHD10 gene used to reduce GC-content in order to facilitate cloning of many patient-derived variants.

B Comparison of HA-tagged CHCHD10 (D10-HA) protein levels in whole cell lysates of HeLa cells transfected with CHCHD10 constructs harboring either the original or the codon-optimized synthetic cDNA.

C Mitochondrial localization of synthetic CHCHD10-HA (D10-HA) was analyzed in transfected HeLa cells by co-staining with a mitochondrial marker (ATP5A1). Scale bar represents 10 μm.

© 2018 The Authors

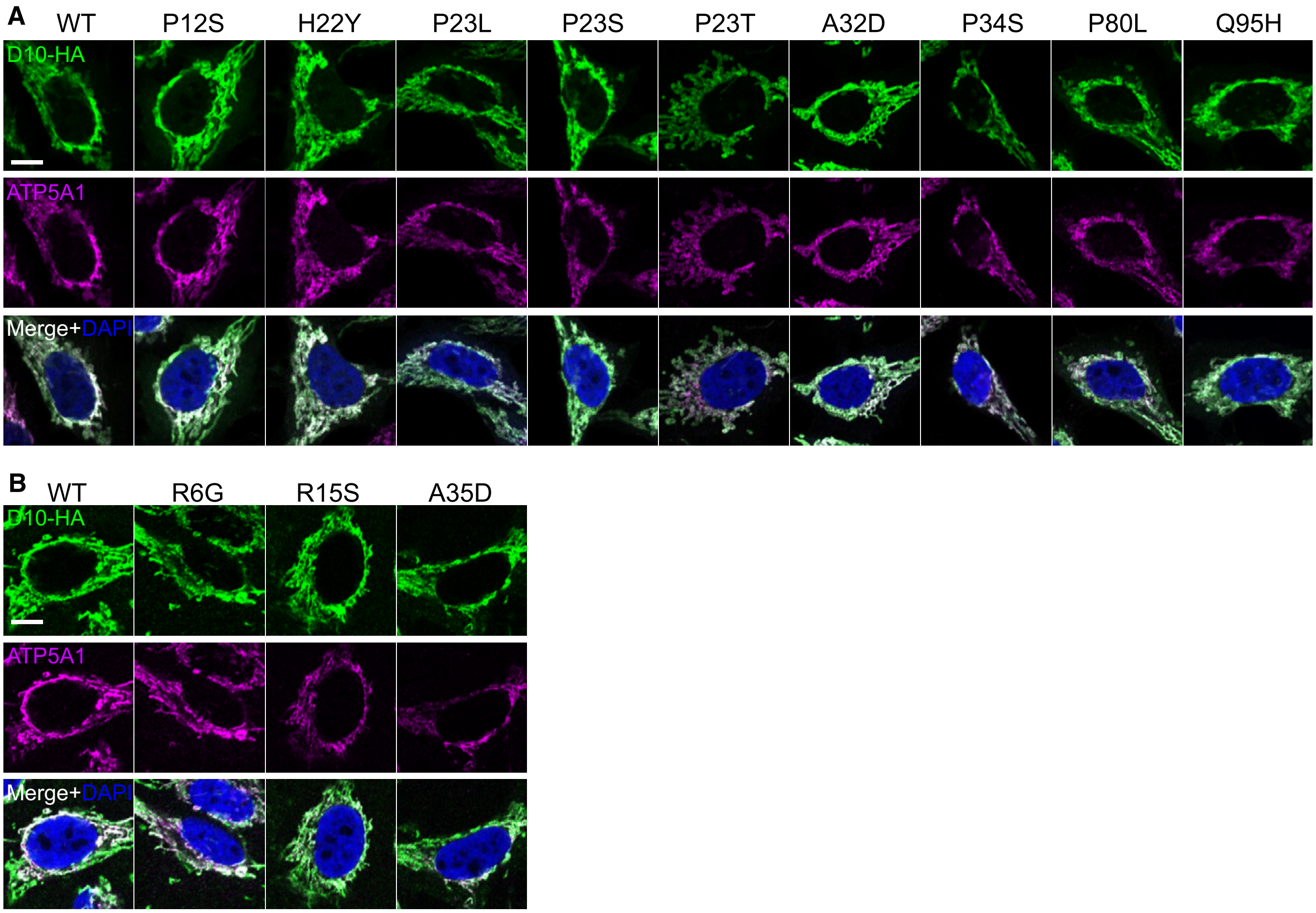

Figure EV3. Subcellular distribution of CHCHD10 patient variants.

A, B HeLa cells were transfected with HA-tagged CHCHD10 (D10-HA) patient variants in two sets (A and B). Immunofluorescence shows expression pattern of CHCHD10-HA (D10-HA) variants compared to the mitochondrial marker ATP5A1. Scale bars represent 10 μm.

© 2018 The Authors

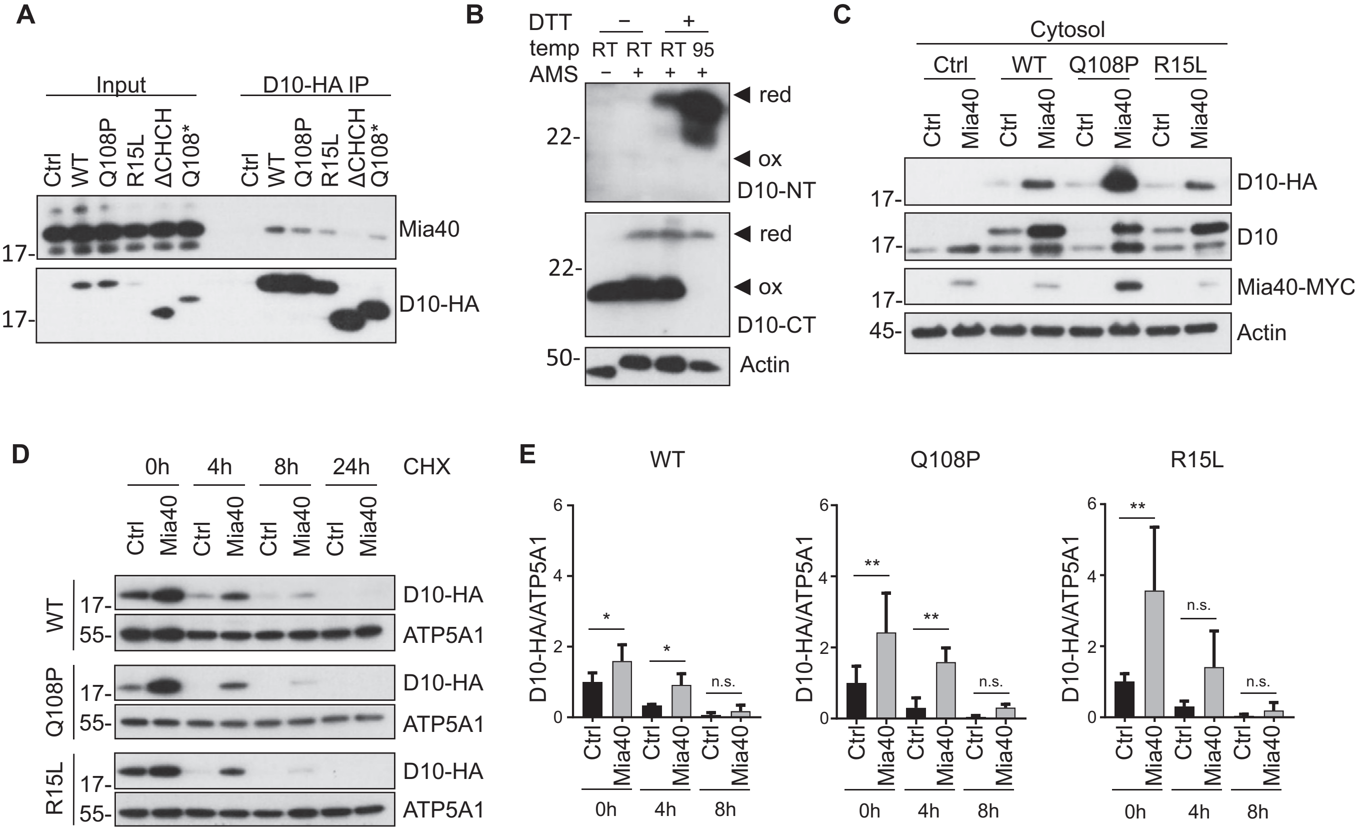

Figure EV4. Mia40 binds and stabilizes CHCHD10.

A Co-immunoprecipitation of Mia40-MYC and CHCHD10-HA (D10-HA) wild-type (WT), variants (Q108P, R15L, ΔCHCH, Q108*), or empty vector (Ctrl) from transfected HeLa cells. Input represents 5% of the whole cell lysate used for immunoprecipitation. Immunoblot of the co-immunoprecipitation was detected with antibodies against Mia40 and HA. Low binding may be explained by the transient interaction of the oxidoreductase Mia40 with its substrate CHCHD10.

B AMS treatment shows disulfide-bond formation of endogenous CHCHD10 in HeLa cells. Note that endogenous CHCHD10, detected with an N-terminal antibody (D10-NT), shows a similar pattern. The C-terminal CHCHD10 antibody poorly detects AMS coupled endogenous CHCHD10 indicating that the epitope overlaps with the cysteine residues. Actin is used as loading control. Note that DTT treatment has no effect on AMS cross-linking of actin, because all its cysteines are reduced in the cytoplasmic environment.

C Immunoblotting shows elevation of CHCHD10 upon Mia40 expression also in cytosolic fractions with indicated antibodies.

D, E Protein stability of HA-tagged CHCHD10 (WT, Q108P, or R15L) upon MIA40 overexpression was measured in HeLa cells treated with cycloheximide (+CHX) and compared to empty vector co-transfection (Ctrl). Cells were harvested after 0, 4, 8, and 24 h. Quantification of CHCHD10-HA (D10-HA) protein levels normalized to ATP5A1. Data are shown as mean ± SD. Two-way ANOVA (with Sidak's multiple comparisons test): n = 4 biological replicates, WT: t = 0 h Ctrl versus Mia40: *P = 0.0146, t = 4 h Ctrl versus Mia40 *P = 0.0168; Q108P: t = 0 h Ctrl versus Mia40: **P = 0.0039, t = 4 h Ctrl versus Mia40 **P = 0.0089; R15L: t = 0 h Ctrl versus Mia40: **P = 0.0014.

 © 2018 The Authors
