# Supplementary figures and images for: A novel CHCHD10 mutation implicates a Mia40‐dependent mitochondrial import deficit in ALS
Source: EMBO Mol Med. 2018 May 22;10(6):e8558. doi: 10.15252/emmm.201708558 (PMC5991575; doi:10.15252/emmm.201708558)

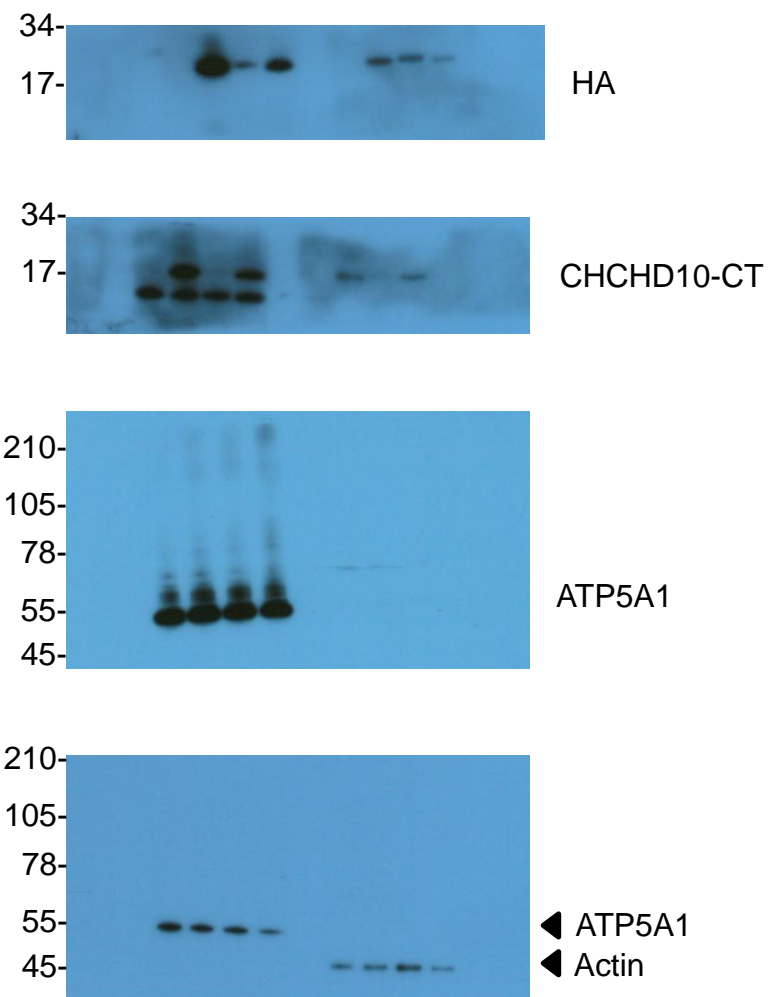

Source Data-Figure 1

Supplement: Supplementary file 3 — Source Data for Figure 1 [file EMMM-10-e8558-s002.pdf]

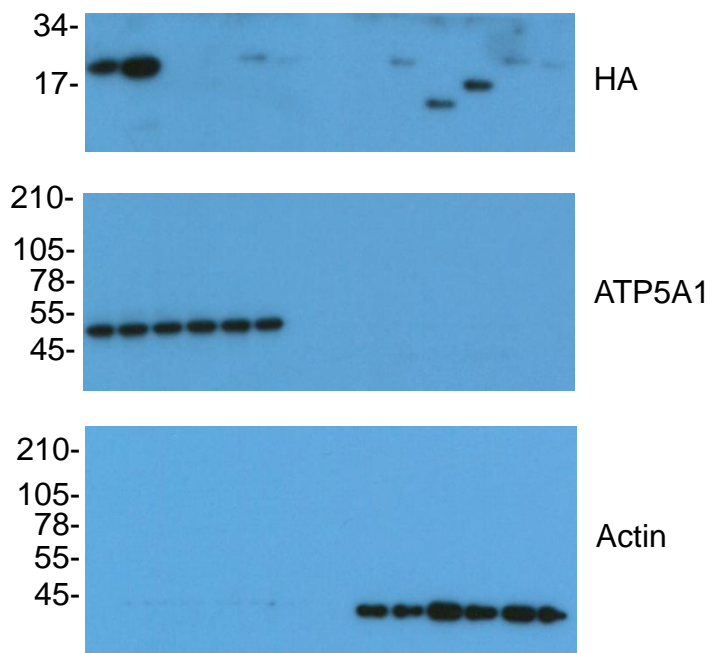

Source Data-Figure 3

Supplement: Supplementary file 4 — Source Data for Figure 3 [file EMMM-10-e8558-s003.pdf]

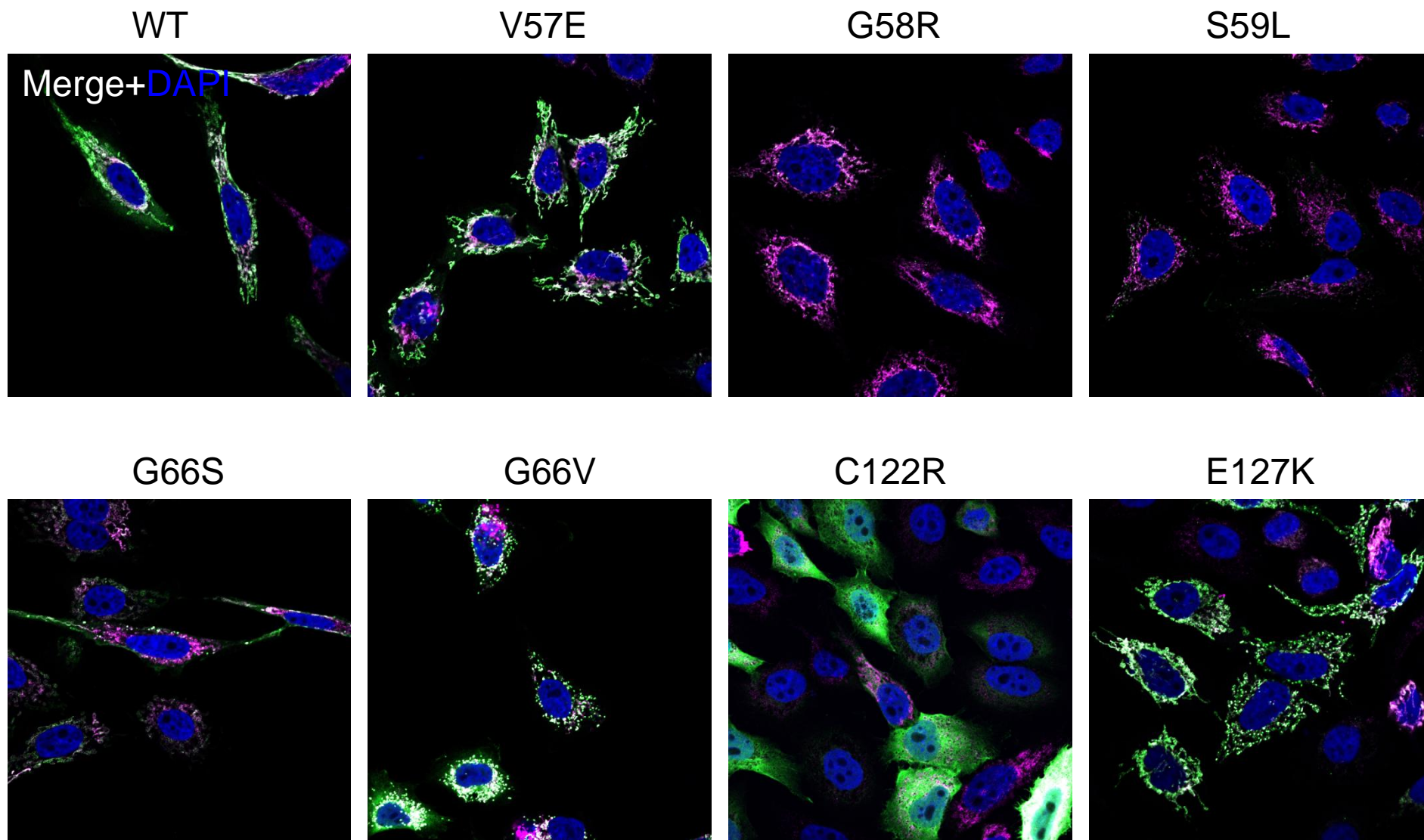

Source Data-Figure 4

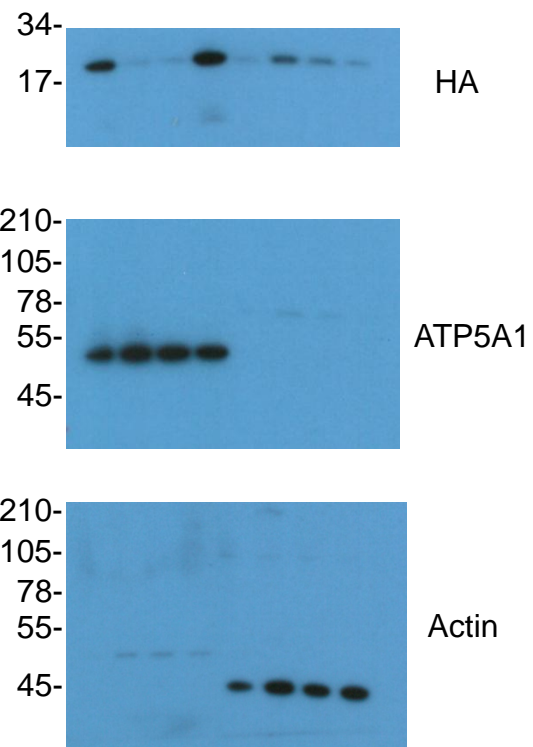

Source Data-Figure 4

Supplement: Supplementary file 5 — Source Data for Figure 4 [file EMMM-10-e8558-s004.pdf]

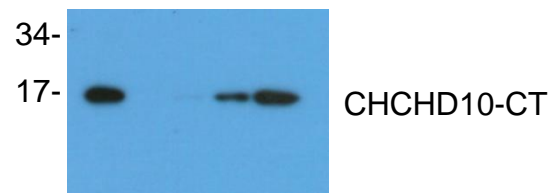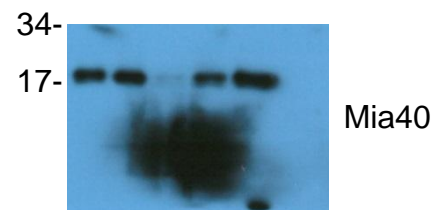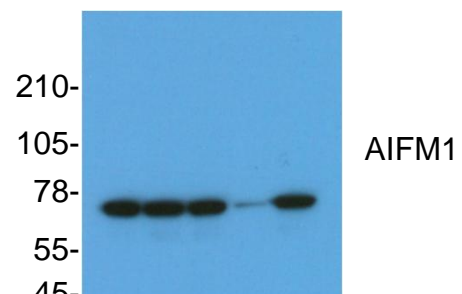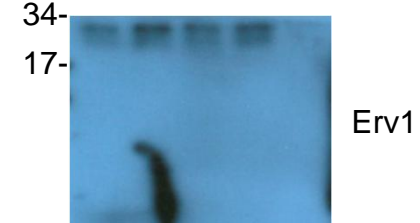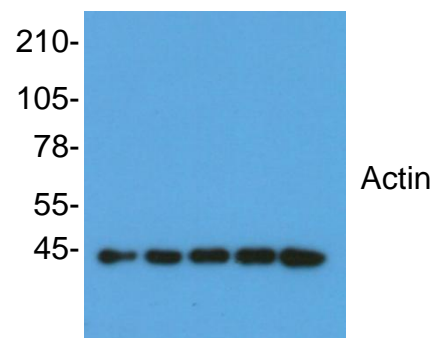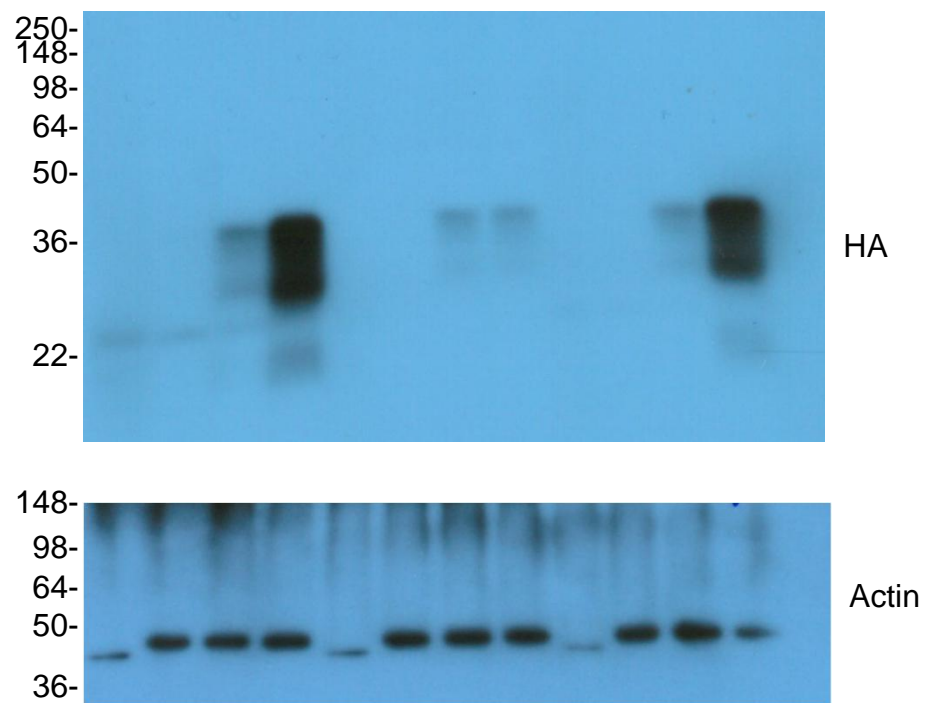

Source Data-Figure 5

Supplement: Supplementary file 6 — Source Data for Figure 5 [file EMMM-10-e8558-s005.pdf]

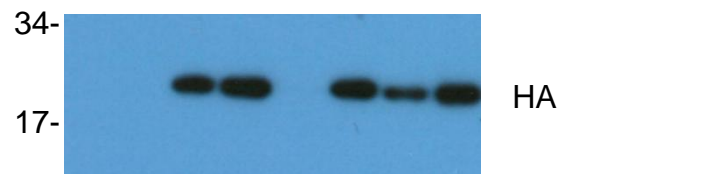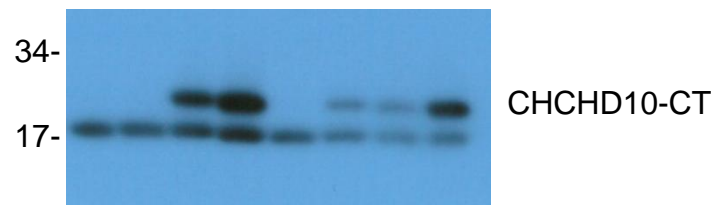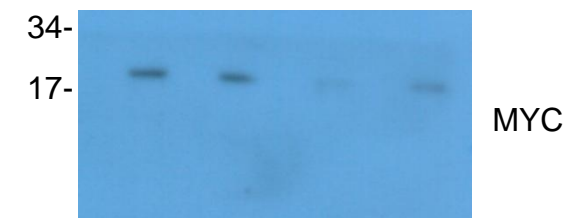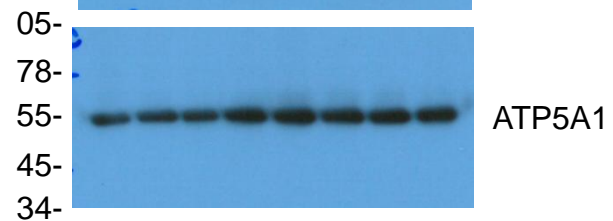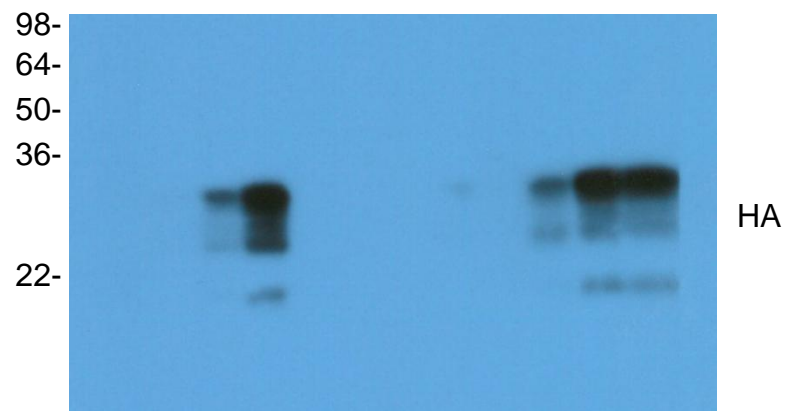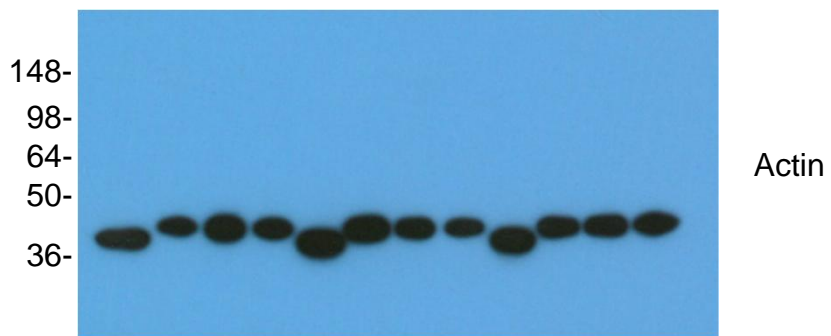

Source Data-Figure 6

Supplement: Supplementary file 7 — Source Data for Figure 6 [file EMMM-10-e8558-s006.pdf]
